# An Intronic Variant of *CHD7* Identified in Autism Patients Interferes with Neuronal Differentiation and Development

**DOI:** 10.1007/s12264-021-00685-w

**Published:** 2021-05-04

**Authors:** Ran Zhang, Hui He, Bo Yuan, Ziyan Wu, Xiuzhen Wang, Yasong Du, Yuejun Chen, Zilong Qiu

**Affiliations:** 1 Institute of Neuroscience, State Key Laboratory of Neuroscience, Key Laboratory of Primate Neurobiology, Center for Excellence in Brain Science and Intelligence Technology, Chinese Academy of Sciences, Shanghai, 200031 China; 2 Shanghai Center for Brain Science and Brain-Inspired Intelligence Technology, Shanghai, 201210 China; 3 University of the Chinese Academy of Sciences, Beijing, 100049 China; 4 Shanghai Mental Health Center, School of Medicine, Shanghai Jiao Tong University, Shanghai, 200030 China

**Keywords:** Autism, *CHD7*, Intronic variant, Inherited variant, *TBR1*

## Abstract

Genetic composition plays critical roles in the pathogenesis of autism spectrum disorder (ASD). Especially, inherited and *de novo* intronic variants are often seen in patients with ASD. However, the biological significance of intronic variants is difficult to address. Here, among a Chinese ASD cohort, we identified a recurrent inherited intronic variant in the *CHD7* gene, which is specifically enriched in East Asian populations. *CHD7* has been implicated in numerous developmental disorders including CHARGE syndrome and ASD. To investigate whether the ASD-associated *CHD7* intronic variant affects neural development, we established human embryonic stem cells carrying this variant using CRISPR/Cas9 methods and found that the level of *CHD7* mRNA significantly decreased compared to control. Upon differentiation towards the forebrain neuronal lineage, we found that neural cells carrying the *CHD7* intronic variant exhibited developmental delay and maturity defects. Importantly, we found that *TBR1*, a gene also implicated in ASD, was significantly increased in neurons carrying the *CHD7* intronic variant, suggesting the intrinsic relevance among ASD genes. Furthermore, the morphological defects found in neurons carrying *CHD7* intronic mutations were rescued by knocking down *TBR1*, indicating that *TBR1* may be responsible for the defects in *CHD7*-related disorders. Finally, the *CHD7* intronic variant generated three abnormal forms of transcripts through alternative splicing, which all exhibited loss-of-function in functional assays. Our study provides crucial evidence supporting the notion that the intronic variant of *CHD7* is potentially an autism susceptibility site, shedding new light on identifying the functions of intronic variants in genetic studies of autism.

**Supplementary Information:**

The online version contains supplementary material available at 10.1007/s12264-021-00685-w.

## Introduction

Autism spectrum disorder (ASD) is known as a specific group of neurodevelopmental disorders characterized by impairments in social interaction and stereotyped behaviors [1]. Although both genetic and environmental factors may contribute to the autistic symptoms, recent genome-wide analyses indicate that genetic composition is the dominant cause of ASD [2–4]. The genetic architecture of ASD is highly heterogeneous as > 100 risk genes have been found [5, 6]. Analysis of *de novo* and inherited rare variations linked to ASD has identified convergent functional themes, such as neuronal development and axon guidance, signaling pathways, and chromatin and transcription regulation [7–9].

Due to the genetic heterogeneity, one strategy is to perform genome sequencing for a large number of ASD families and identify risk genes more comprehensively. As a result of the increasing resolution and decreasing cost of DNA sequencing technology such as karyotyping, microarrays, whole-exome sequencing, and whole-genome sequencing, nearly 20% of ASD cases can be explained by identifiable genetic causes, while the rest remain unclear. So far, most efforts have been focused on rare deleterious *de novo* single-nucleotide variants (likely gene-disrupting variants) on coding regions or splicing sites. However, multiple common inherited variants are clearly suspect factors for ASD [10]. Since inherited variants are also present in unaffected parents, identifying the causal relationship between inherited variations and ASD pathogenesis requires neurobiological evidence as well as genetic evidence [11, 12].

The current largest genetic sequencing projects for ASD cohorts are from Europe and the USA, which are mostly composed of Caucasian, Latino, Ashkenazi Jewish, and African–American populations. Due to the genetic and geographical differences between Caucasians and Asians, the potential genetic causes for Asians population may incompletely overlap with those in the western world. Considering the vast population of China, there is no doubt that a large population of individuals with ASD exist in the Chinese population. Identification of the ASD risk genes based on the Chinese population is not only critical to provide precise diagnoses and specific interventions for Chinese ASD patients, but also to provide an important missing piece for a comprehensive and in-depth understanding of autism [13].

Therefore, we set out performing whole-exome sequencing for Chinese ASD probands along with their parents to identify mutations that do not exist in common databases and to explore the new pathogenesis of ASD.

## Materials and Methods

### Ethics Statement

We obtained assent from the Institutional Review Board (IRB), Shanghai Mental Health Center of Shanghai Jiao Tong University (Federalwide Assurance Number: 00003065; IRB Organization Number: 0002202). Dr. Yi-Feng Xu approved and signed our study with ethical review number 2016–4. Written informed consent was given by parents since all patients were minors. All participants were screened using the appropriate protocol approved by the IRB of Shanghai Mental Health Center, Shanghai Jiao Tong University School of Medicine.

### Participants

A total of 168 core families with probands diagnosed with ASD were recruited from among the outpatients in the Department of the Child and Adolescent Psychiatry, Shanghai Mental Health Center. All patients were diagnosed on the basis of the fifth edition of the Diagnostic and Statistical Manual of Mental Disorders. All patients were Han Chinese and their ages ranged from 2 to 18 years.

### Human H9 Embryonic Stem Cells (hESCs)

hESCs (H9, line WA09 (WiCell), passages 20–40) were cultured on a feeder layer of irradiated mouse embryonic fibroblasts in a humidified incubator with 5% CO_2_ at 37 °C. The hESC medium containing DMEM/F12 (Gibco), 20% Knock Serum Replacement (Gibco, 10828010), 0.1 mmol/L beta-mercaptoethanol (Sigma), 1% (v/v) Non-Essential Amino Acids (NEAA, Life Technologies), and 0.5% (v/v) GlutaMAX (Life Technologies). The medium was changed every day with 10 ng/mL of basic fibroblast growth factor (PeproTech). hESCs were passaged every 7 days. hESCs with an intronic mutation (8-61757392-C-T) were transformed from H9 hESCs by introducing an intronic mutation and two synonymous mutations by homology-mediated end joining-based targeted integration using CRISPR/Cas9. The editor vector (Addgene #48138) containing the small guiding RNA (sgRNA) that targeting exon sequence of *CHD7*, together with the donor vector containing a 1.6 kB homologous sequence of *CHD7* and carrying an intronic mutation and two synonymous mutations, were transfected into H9 hESCs by liposome. Single GFP^+^ cells were isolated using flow cytometry and cultured. The cells with an intronic point mutation were identified by DNA sequencing.

### Human Embryonic Kidney (HEK) 293 Cells

HEK293 cells were purchased from Cell Bank of the Chinese Academy of Sciences (Shanghai, China). HEK293 cells with the intronic mutation (8-61757392-C-T) were transformed from HEK293 cells by introducing an intronic mutation and two synonymous mutations by homology-mediated end joining-based targeted integration using CRISPR/Cas9. The editor vector (Addgene #48138) containing the small guiding RNA (sgRNA) that targeting exon sequence of *CHD7*, together with the donor vector containing a 1.6 kB homologous sequence of *CHD7* and carrying an intronic mutation and two synonymous mutations, were transfected into H9 hESCs by liposome. Single GFP^+^ cells were isolated using flow cytometry and cultured. The cells with the intronic point mutation were identified by DNA sequencing. HEK293 synonymous control cells were similar to HEK293 intronic mutation cells with two synonymous mutations but without the intronic mutation. All the HEK293 cells were cultured in DMEM (Gibco/Life Technologies) with 10% FBS (Gibco/Life Technologies) at 37 °C in a 5% CO_2_ incubator (Thermo Scientific Heraeus).

The primers for cell genotypes were as follows:

*CHD7*-forward: GAAGTTCACAGGAGCCAGAG

*CHD7*-reverse: CAGAAAGTAGAATGGTGATTGCCAG

### Primary Cultures of Cortical Neurons

Mouse cortical neurons were cultured from E14.5 C57BL/6J of either sex. Cerebral cortices were dissected, dissociated, and cultured in 0.5 mL/well Neurobasal medium (Gibco, 21103-049) with 2% B27 (Gibco, 17504-044) and 2 mmol/L Glutamax-I (Gibco, 35050-061) on Lab-Tek II Chamber Slides (Thermo Fisher Scientific, 154941) at 100,000 cells/cm^2^. For the axon and dendrite experiments, the neurons were transfected by Lipo3000 (Invitrogen, L3000075) with 0.9 μg vector, following the Lipofectamine^TM^ 3000 Reagent Protocol, 24 h after plating. After transfection, the cultures were fed with new medium every 2 days.

### Plasmid Construct

The gene editor vector was an sgRNA targeting sequence cloned in CRISPR/Cas9 vector (Addgene #48138). The donor vector was the homologous arm cloned in pUC57. The vector that expressed GFP was FUGW (Addgene #14883). The control expression vector was FUGW with GFP removed. The vector expressing CHD7 was a gift from Prof. Wei-Jun Feng (Institutes of Biomedical Sciences Fudan University, Shanghai, China). The vectors expressing alternative forms of *CHD7* (exons 22–23 deletion and exons 22–23 duplication) were modified by enzyme ligation and homologous recombination from the vector expressing *CHD7*. The shRNAs for mouse *Chd7*, human *CHD7*, and human *TBR1* were cloned into the FUGW-H1 vector (Addgene #25870); the shRNA for control was *DsRed*.

The shRNA sequences were as follows:

mouse *Chd7*: GCAGCAGCCTCGTTCGTTTAT

human *CHD7*: GCAGCAGTCTCGTCCATTTAT

human *TBR1*: GCCTTTCTCCTTCTATCATGC

*DsRed*: AGTTCCAGTACGGCTCCAA

### Whole-Exome Sequencing

DNA was extracted from the peripheral blood of patients and their parents using the DNeasy Blood & Tissue Kit (Qiagen, 69506, Germany), following the manufacturer’s instructions. For each sample to be sequenced, individual library preparation, hybridization, and capture were performed following the protocol of the Agilent SureSelect capture kit (V5) or the IDT XGen Exome Research Panel. Sequencing was performed on an Illumina HiSeq X-10 instrument (Illumina) following the manufacturer’s protocol (HiSeq X-10 System User Guide).

### Variation Identification by Sanger Sequencing

Based on the data from whole-exome sequencing (WES), all families with probands carrying *CHD7* variations were selected for Sanger sequencing to validate whether the variations were *de novo* or inherited from parents. The primers for Sanger sequencing were as follows:

*CHD7*-forward: CCAGGGTTAGCTTTGTGGGT

*CHD7*-reverse: TGGCTTTGTGACCCTGTAGC

### RNA Isolation and Reverse Transcription

Each group of cells was dissociation in 1 mL TRIzol (Invitrogen, 15596018). Total RNA was isolated using the method in the user guide for TRIzol^TM^ Reagent. Reverse transcription used the Reverse Transcriptase M-MLV kit (TaKaRa, D2639B). One microgram of total mRNA and 50 nmol oligo dT were used as primers in the reverse transcription.

### Quantitative Real-Time RT-PCR (qPCR)

For qPCR analysis, the gene expression of cDNA samples was analyzed using SYBR green (Toyobo, QPK-201). The qPCR program was three steps with melting as follows: 95 °C denaturation for 10 min, followed by 40 cycles of 95 °C for 10 s, 60 °C for 15 s, and 72 °C for 20 s. The RNA level was calculated and standardized using the ΔCt method and the GAPDH expression level as control.

The primers for qPCR were as follows:

*CHD7* exon 19-forward: ACGAAAAGGGGCCTATGGTG

*CHD7* exon 20-reverse: TTCAGCCTTCTTAGCCCACT

*CHD7* exon 25-forward: TCCCTGAACCTTTCCATGCT

*CHD7* exon 26-reverse: TCCCTGAACCTTTCCATGCT

*CHD7* exon 35-forward: ATGGCTGAAGCTGCACCCTA

*CHD7* exon 38-reverse: AGGCGGTCAAACATCGACTC

*CHD7* intronic retention-forward: TGGAGAAGAATCTGCTTGTCTATGGG

*CHD7* intronic retention-reverse: TCTGGGCTTTCACCTTCTTT

*CHD7* exon 22–23 deletion-forward: AATCTGCTTGTCTATGGGGTCC

*CHD7* exon 22–23 deletion-reverse: TCCCTGAACCTTTCCATGCT

*CHD7* exon 22–23 duplication-forward: CAACCATTCCGGTTTGTCAGC

*CHD7* exon 22–23 duplication-reverse: TCTGGGCTTTCACCTTCTTT

*TBR1*-forward: GACTCAGTTCATCGCCGTCA

*TBR1*-reverse: TGCTCACGAACTGGTCCTG

*GAPDH*-forward: CATCGCTCAGACACCATGGG

*GAPDH*-reverse: CCTTGACGGTGCCATGGAAT

*Chd7*-forward: TCCACATTTGCTAAGGCCAG

*Chd7*-reverse: TTCAGCCTTCTTAGCCCACT

*Gapdh*-forward: GTGAAGGTCGGTGTGAACGG

*Gapdh*-reverse: CGCTCCTGGAAGATGGTGAT

### Differentiation of Dorsal Forebrain Glutamate Neurons

hESC colonies were cultured with daily medium changes until they reached approximately 80% confluence. Then, the colonies were detached from the feeder layer by digestion with dispase (Life Technologies), and re-suspended in hESC medium for 4 days to form embryoid bodies (EBs). For neural induction, the EBs were cultured in neural induction medium [DMEM/F12, 1% (v/v) N2 supplement, 5% (v/v) B27 without RA, 1% (v/v) NEAA, all from Life Technologies (NIM)] supplemented with SB-431542 (2 μmol/L, Stemgent) and DMH-1 (2 μmol/L, Tocris) for 3 days. The EBs were then attached to a 6-well plate in NIM supplemented with 5% fetal bovine serum. The cells were fed with NIM every other day until neural tube-like rosette formation at around day 16. Then, the rosettes were blown off using a 1-mL pipette and cultured in suspension. After 2 days, the cell clusters formed neurospheres, and then the medium was changed every other day. On day 26, the neurospheres were digested into single cells using accutase, and seeded at ~ 40,000 cells/cm^2^ on coverslips pre-coated with Matrigel. After 5 h–6 h, neuronal differentiation medium [neural basal media, 1% (v/v) N2, BDNF (10 ng/mL, PeproTech), GDNF (10 ng/mL, PeproTech), cAMP (1 μmol/L, Sigma), IGF-I (10 ng/mL, PeproTech), and AA (200 μmol/L, Sigma)] was added to the wells, and the medium was changed weekly.

### Immunohistochemical Staining

Cultured cells were washed with PBS for 5 min, fixed in 4% PFA at room temperature for 30 min, then washed twice with PBS every 10 min. The cells were blocked with 5% BSA and 0.3% TritonX-100 in PBS at room temperature for 2 h, then incubated overnight at 4 °C with primary antibody in 3% BSA and 0.1% TritonX-100 in PBS. After washing 3 times with PBS every 10 min, they were incubated at room temperature with secondary antibody and DAPI in PBS for 2 h, then washed 3 times with PBS every 10 min.

The primary antibodies and dilutions were as follows:

Anti-SOX2 (R&D, AF2018, 1:500)

Anti-PAX6 (DSHB, AB-528427, 1:10)

Anti-TUJ1 (Sigma, T8660, 1:5000)

Anti-KI67 (Abcam, ab15580, 1:800)

Anti-CHD7 (CST, #6505, 1:1000)

Anti-GFP (Abcam, ab6673, 1:400)

Anti-MAP2 (Millipore, MAB3418, 1:1000)

Anti-SMI312 (Biolegend, 837904, 1:1000)

Anti-TBR1 (Abcam, ab31940, 1:1000)

Anti-CTIP2 (Abcam, ab18465, 1:200)

Hoechst (Life-tech/3570, 1:2000)

DAPI (Sigma, D9542, 1:1000)

### Western Blot

SDS-polyacrylamide gradient gel (4%–20%) was used in the Western blot; the electrophoresis program was 80 V for 30 min and then 120 V for 180 min. Proteins were transferred onto Immobilon polyvinylidene difluoride membranes (Millipore) for 210 min at 200 mA. The membrane was blocked by TBST (50 mmol/L Tris-HCl, pH 7.5, 150 mmol/L NaCl, and 0.1% Tween 20) with 5% BSA for 2 h at room temperature, incubated overnight at 4 °C with primary antibody in 3% BSA, then washed 3 times with TBST every 10 min. The membrane was treated with secondary antibody for 2 h at room temperature, then washed 3 times with TBST every 10 min. The reaction was analyzed using imaging film.

The primary antibodies and dilutions were as follows:

Anti-CHD7 (CST, #6505, 1:1000)

Anti-GAPDH (ab8245, 1:5000)

### Analysis of Dendrites and Axons

About 40–50 GFP-positive (GFP^+^) neurons were picked up randomly from each group. The searcher was blinded until statistical analysis was completed. The images were analyzed using Fiji software: all dendritic branches and secondary branches, the longest axon and secondary branches, and the total length of all neurites were taken into account. At least three independent experiments were performed,

### Alternative Splicing Analysis

cDNA was segmentally amplified by PCR. The products were separated by agarose gel electrophoresis and retrieved, then ligated with pGEM-T Easy Vector (Promega). The ligation products were transformed into *Escherichia coli* Top10 and monoclonal culture. At least 40 monoclonals were sequenced per sample.

### Transcriptome Analysis (RNAseq)

For RNA-sequencing, total RNA was extracted and subsequently a sequencing library was prepared using the Illumina TrueSeq Total RNA Sample Prep Kit and sequenced on the Illumina Hi-Seq 2000. The clean reads were aligned to 9606 (NCBI Taxonomy ID) genome (version: GRCh38) using Hisat2. We applied HTseq to calculate the counts of genes. Reads/Fragments Per Kilobase Million Reads was used to standardize the expression data. We applied the DEseq2 algorithm to filter the differentially-expressed genes, then we filtered fold-change (FC) and false discovery rate (FDR) under the following criteria: (a) log_2_(FC) > 0.585 or < − 0.585; (b) FDR < 0.05. For Gene ontology (GO) analysis, we downloaded the GO annotations from NCBI (http://www.ncbi.nlm.nih.gov/), UniProt (http://www.uniprot.org/) and GO (http://www.geneontology.org/). Fisher's exact test was applied to identify the significant GO categories and FDR was used to correct the *P*-values.

### Statistical Analysis

Statistical tests were carried out using GraphPad Prism 6 (GraphPad Software, Inc., RRID:SCR_002798). Two-tailed Student's *t*-test was used for sample pairs, one-way ANOVA followed by Tukey's multiple comparison tests was used for 3 or more groups. Data distribution was tested by the Kolmogorov–Smirnov and Shapiro–Wilk tests in SPSS software (IBM, RRID:SCR_002865). The data distribution was normal. Results are shown as the mean ± SEM, and “*n*” represents either the number of neurons (for morphological analysis) or the number of repeated experiments (for qPCR and RNAseq). Mouse cortical neurons were independently obtained at least 3 times from 3 different litters. Stem cell differentiation was carried out independently in at least 3 batches. All data analyses were performed blinded to the experimental conditions. All conditions statistically different from the control are indicated as: *P < 0.05; **P < 0.01; ***P < 0.001; ****P < 0.0001. If the data were not in the 95% confidence interval of the group, they were excluded.

## Results

### An Intronic Variant of the *CHD7* Gene Found in Chinese Patients with ASD Leads to Down-Regulation of *CHD7* mRNA

**Fig. 1.**
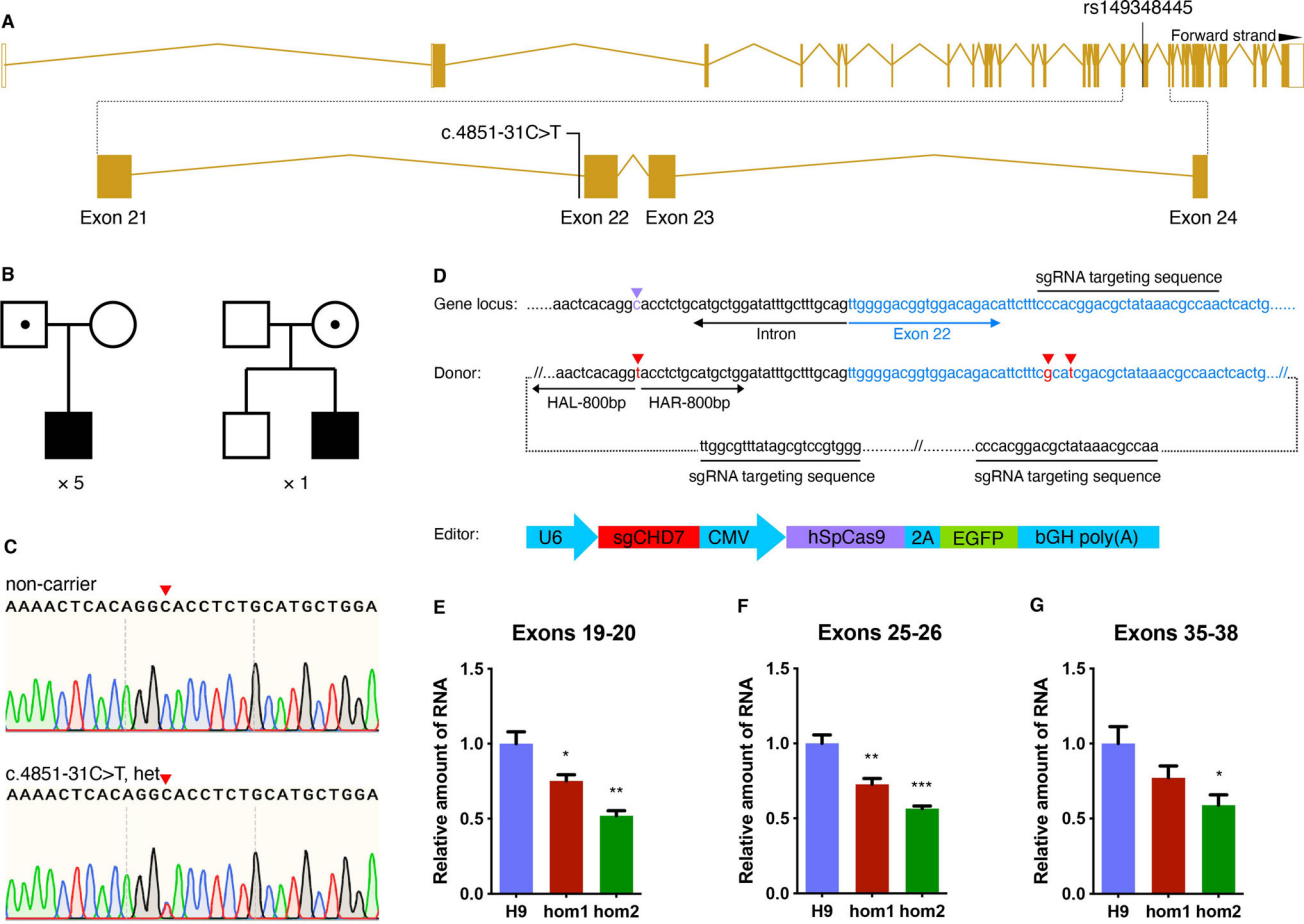
An intronic variation of *CHD7* found in Chinese ASD patients leads to down-regulation of *CHD7* mRNA. **A** Position of the intronic variation in the genomic structure of the *CHD7* gene. **B** Genogram of the six families with probands carrying the *CHD7* variation (blank, non-carrier; blank with dot, asymptomatic carrier; solid, affected carrier; squares and circles represent males and females, respectively). **C** Sanger sequencing to verify the intronic variation in the *CHD7* gene (red arrowheads, variant base). **D** Schematic of gene editing (purple arrowhead, normal base; red arrowheads, introduced mutant bases; HAL/HAR, left/right homology arm). **E**–**G** mRNA levels assessed by RT-qPCR for *CHD7* in cell lines carrying homozygous mutants (hom1 and hom2) with primers located in exons 19–20 (**E**); exons 25–26 (**F**); and exons 35–38 (**G**). Values represent the mean ± SEM (*n* = 3, *P < 0.05, **P < 0.01, ***P < 0.001, ****P < 0.0001; one-way ANOVA). See also Fig. S1.

After whole-exome sequencing for 167 ASD probands and their parents from Shanghai Mental Health Center, we identified an intronic variation in the *CHD7* gene (NM_017780.3:c.4851-31C>T, het) in 6 probands (Fig. 1A). Variants of five probands were inherited paternally, and one variant was inherited maternally and his non-carrier brother was unaffected (Fig. 1B). Sanger sequencing was carried out to confirm the presence of the variant (Fig. 1C).

When we examined the frequency of this variant (rs149348445) in various populations in the gnomAD database, we surprisingly found that this variant only existed in East Asia populations as a common variant with relatively low frequency (0.39%), and not in Caucasian or other populations. However, this variant had a nearly 10-times enriched frequency (3.6%, 6/167) in our ASD cohort, strongly suggesting that this variant is implicated in ASD.

The CHD7 protein belongs to the CHD family of chromatin remodelers and catalyzes the translocation of nucleosomes along DNA in chromatin [14]. Mutations of the *CHD7* gene are the major cause of CHARGE syndrome, which is characterized by coloboma of the eye, heart defects, atresia of the choanae, retardation of growth and development, genital abnormalities, and ear abnormalities [15]. Children with CHARGE syndrome frequently exhibit autistic-like deficits in vocalization, social responsiveness, and repetitive behaviors, suggesting that *CHD7* has a direct impact on autism [16].

Due to the high variability of non-coding regions between rodents and humans, the mouse *Chd7* gene does not contain similar sites with which we could make mouse models to mimic the condition. Therefore, to investigate whether this intronic variant affects the expression of the *CHD7* gene, we set out to perform mutagenesis in hESCs.

The point mutation was introduced into hESCs (H9) by homology-mediated end joining-based targeted integration with CRISPR/Cas9 technology [17]. Two synonymous mutations were also introduced into the sgRNA target region within exon 22 to avoid unwanted digestion by Cas9 after recombination (Fig. 1D). Two sub-clones (hom1-H9-hESCs and hom2-H9-hESCs) were successfully established, which carried the intronic mutations in a homozygous manner (hom; Fig. S1A). Unfortunately, heterozygous mutations (het) failed to be established.

In order to eliminate the risk of off-target effects, the sgRNA used was not matched with any regions outside the target. Nevertheless, we verified the top five predicted high risks (Fig. S1B) and no off-targets were found.

To determine whether the point mutation affected the expression of *CHD7*, we analyzed the mRNA expression using real-time qPCR with multiple primer pairs amplifying exons adjacent to the intronic variant site and the mRNA terminus. We found that the relative amount of *CHD7* mRNA was down-regulated in both cell lines carrying homozygous mutants (hom1-H9-hESCs and hom2-H9-hESCs) compared to H9 wild-type cells, using primer pairs amplified upstream (exons 19–20) or downstream exons (exons 25–26) of the point mutation, as well as a primer pair amplifying the 3’ end of mRNA (exons 35–38) (Fig. 1E–G). This evidence indicated that the intronic variant (ch8-61757392-C-T) in the *CHD7* gene affects the expression of *CHD7* in human cells.

### Intronic Variation of *CHD7* Delays Neuronal Differentiation of Human ESCs

*Chd7* has been implicated in adult neurogenesis and the neural differentiation of cerebellar granule cells in mice [18, 19]. In order to study whether the intronic variation of *CHD7* found in autistic patients affects neuronal differentiation, we differentiated hESCs into dorsal forebrain glutamate neurons according to an established protocol (Fig. S2A).

**Fig. 2.**
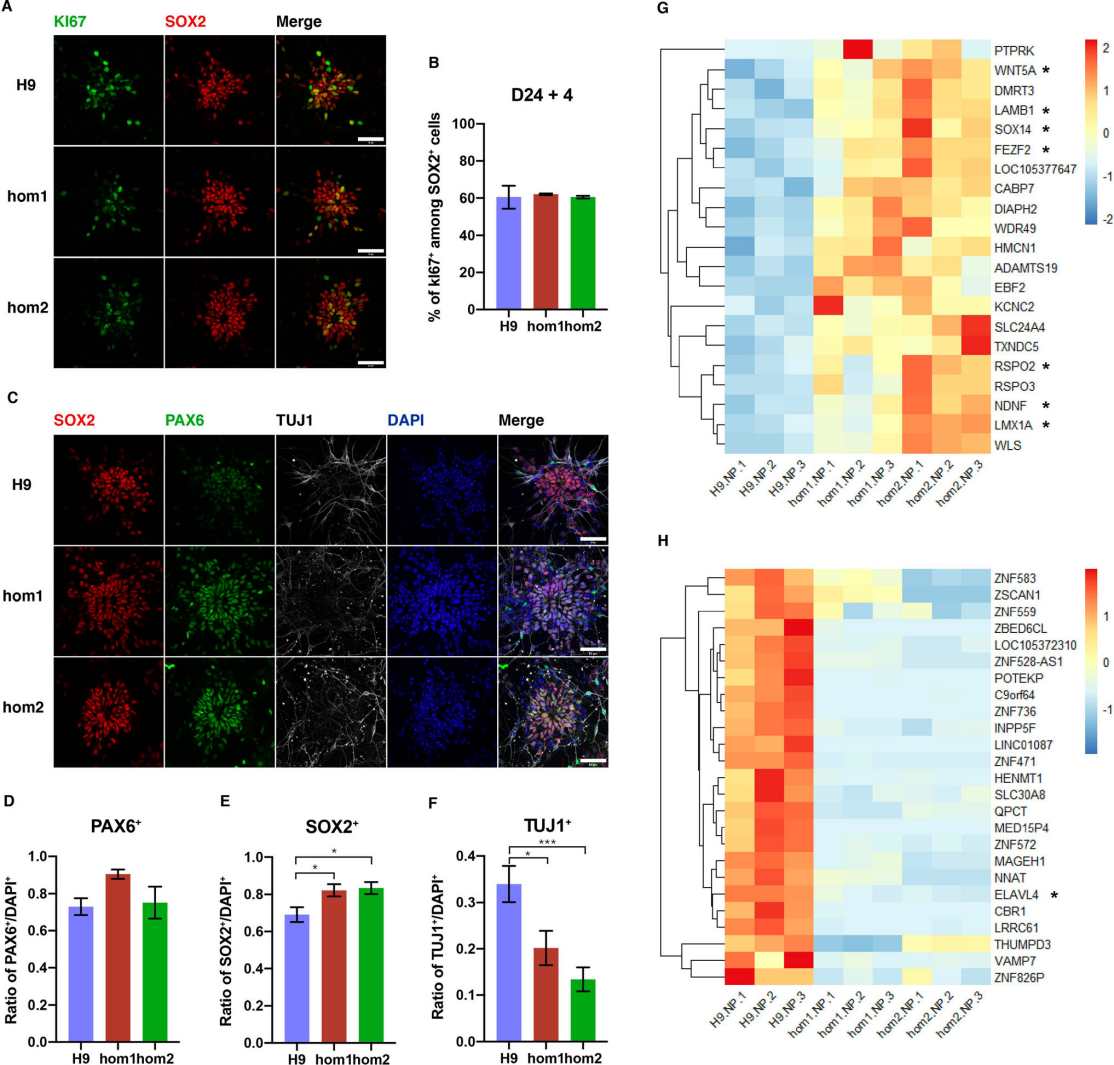
Intronic variation of *CHD7* delays neuronal differentiation of hESCs. **A** Confocal images of differentiated human neural precursor cells (H9 control, hom1, and hom2) on D28 co-immunostained for Ki67 and SOX2 (scale bars, 50 μm). **B** Proportions of Ki67-positive cells among SOX2-positive cells (at least 1000 cells in 8 fields were analyzed for each group). **C** Confocal images of differentiated human neural precursor cells (H9 control, hom1, and hom2) on D28 co-immunostained for SOX2, PAX6, TUJ1, and DAPI (scale bars, 50 μm). **D**–**F** Statistical ratios of PAX6 (**D**), SOX2 (**E**), and TUJ1 (**F**) to DAPI (at least 1,000 cells in 8 fields were analyzed for each group; *P < 0.05, **P < 0.01, ***P < 0.001, ****P < 0.0001; one-way ANOVA). **G**, **H** Heatmaps showing significantly up-regulated genes (**G**) and down-regulated genes (**H**) (fold change > 1.5, FDR < 0.05) in differentiated neural precursor hom1 and hom2 cells compared to H9 control on D26 (color scale on right; *genes involved in neural differentiation and development; see also Fig. S2).

On day 28 (D28) of neural differentiation, we first examined whether the proliferation of NPCs was affected by the intronic variant of *CHD7*. We performed immunostaining using antibodies against the proliferation marker KI67 and the neural stem cell marker SOX2. We found that the percentage of KI67-positive cells among SOX2-positive cells were similar in NPCs derived from wild-type H9 cells and the two cell lines (hom1-H9-hESCs and hom2-H9-hESCs) carrying homozygous variants (Fig. 2A, B), suggesting that the intronic variation has no effect on the proliferation of human NPCs.

In order to further examine the process of neural differentiation, we performed immunostaining for SOX2 (an early neural stem cell marker), PAX6 (an intermediate neural stem cell marker), and TUJ1 (a neuronal marker) on D28 (Fig. 2C). We first found that most of the neuronal cells derived from wild-type or mutant hESCs expressed the dorsal forebrain progenitor marker PAX6 in a similar proportion, suggesting that the intronic mutation of *CHD7* did not affect the dorsal forebrain progenitor differentiation of hESCs (Fig. 2D). Interestingly, there were more SOX2-positive cells in the culture derived from both mutant hESC lines than in those from wild-type hESCs (Fig. 2E). In contrast, the proportion of TUJ1-positive cells derived from mutant hESCs was much lower than that in wild-type hESCs (Fig. 2F). These results are consistent with the previous report that a lack of Chd7 in mouse neural stem cells causes delayed neural differentiation [18].

Taken together, this evidence strongly suggests that the increase of SOX2-positive cells in hESCs-derived neurons carrying the intronic variant is most likely due to the delayed differentiation of forebrain stem cells, rather than increased proliferative capacity.

Chd7 plays a critical role in chromatin remodeling, and it has been shown that loss of Chd7 leads to dramatic changes in gene expression [19]. To further determine the molecular mechanism by which CHD7 regulates neural differentiation, we performed RNA-seq with RNA collected from neural precursor cells at D26 after the initiation of neural differentiation. We found that the expression of numerous genes changed significantly in NPCs carrying *CHD7* intronic variants comparing to wild-type NPCs (Fig. 2G, H). Altered genes were involved in biological processes including dopaminergic neuron differentiation, dentate gyrus development, and the Wnt signaling pathway (Fig. S2B). Genes closely associated with neural differentiation and development are of great interest, (marked with an asterisk in Fig. 2G, H) and we carried out real-time qPCR to verify the expression change (Fig. S2C–J). Among them, *LMX1A* is required for proper ear histogenesis and morphogenesis [20], and loss of hearing is a pivotal defect of the CHARGE syndrome. *WNT5A*, a critical gene in the Wnt signaling pathway, favors bone marrow MSC differentiation into osteoblasts by inhibiting the function of activated PPARγ through complex formation between NLK, SETDB1, and CHD7 [21]. *LAMB1* is one of the risk genes for ASD [22]. This evidence indicates that the intronic variant in the *CHD7* gene leads to the dysregulation of a series of critical genes that are implicated in neural developmental disorders, and the effect is similar to that caused by gene deletion.

### Intronic Variation of *CHD7* Impairs Neurite Development and Dendritic Morphology

Cortical glutamatergic neurons comprise the major excitatory network in the central nervous system [23]. Glutamatergic neurons play critical roles in controlling cognition, emotion, language, and motor function. Dysfunction of cortical glutamatergic neurons may be relevant to autism [24]. Several of the known genetic disorders associated with autism have important implications for glutamatergic deficits in the disorder.

It has been reported that the development of newborn neurons in the subgranular zone of adult *Chd7*-null mice is severely compromised, showing less complex dendritic morphology than wild-type newborn neurons [18]. In order to study whether the intronic variation of *CHD7* found in autistic patients affects neuronal development, we differentiated hESCs into dorsal forebrain glutamate neurons using an established protocol (Fig. S2A). The cortical deeper-layer markers of glutamatergic neurons, TBR1 and CTIP2, were observed on D40 (Fig. S3).

**Fig. 3.**
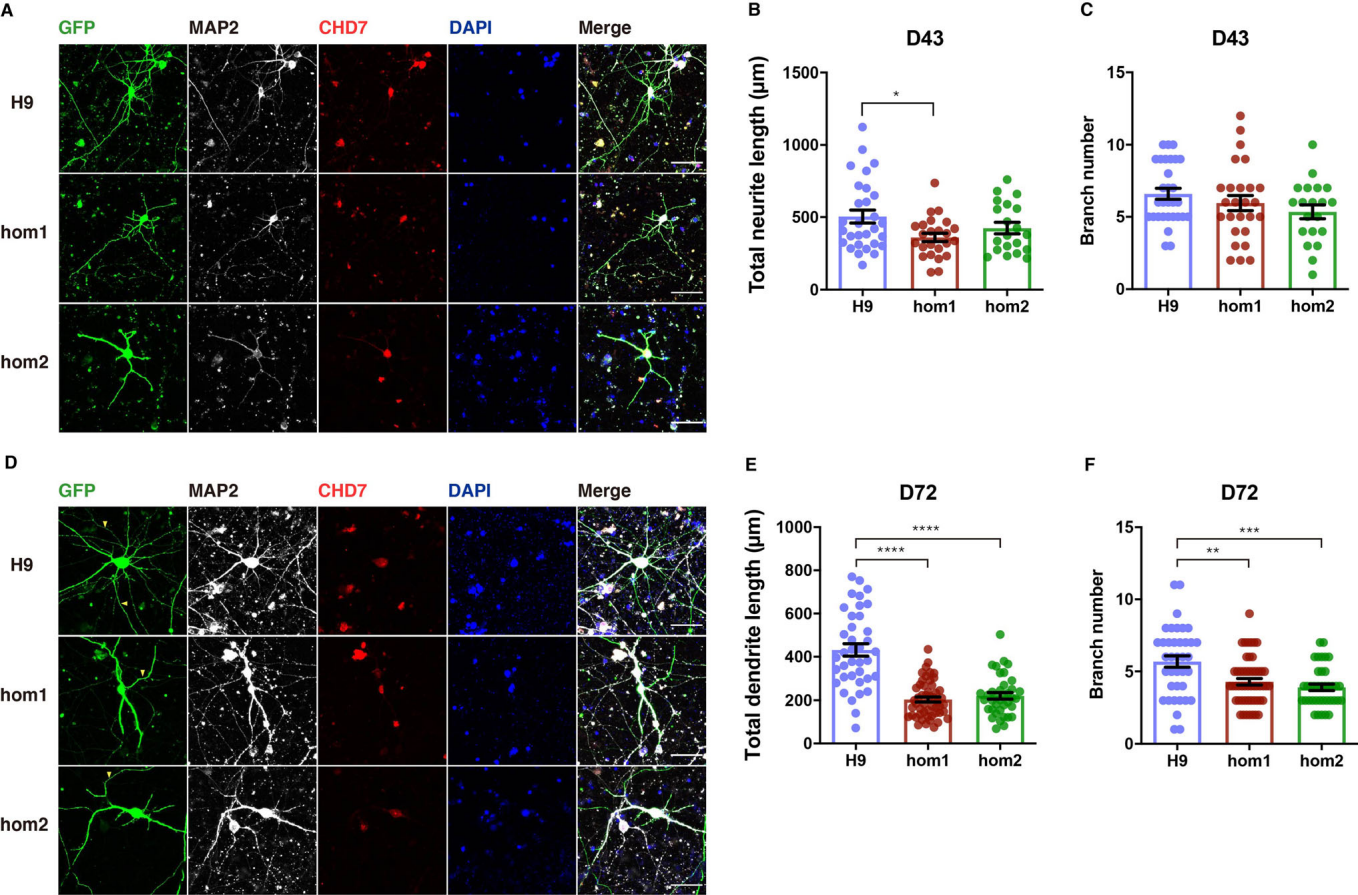
Intronic variation of *CHD7* impairs neurite development and dendritic morphology. **A** Representative images of differentiated neurons (H9 control, hom1, and hom2) on D43 transfected with GFP, co-immunostained for GFP, MAP2, CHD7, and DAPI (scale bars, 50 μm). **B**, **C** Quantification of total neurite length (**B**) and branch number of differentiated neurons (**C**) (H9 control hom1, and hom2) on D43 (25–30 neurons were analyzed for each group; *P < 0.05, **P < 0.01, ***P < 0.001, ****P < 0.0001; one-way ANOVA). **D** Representative images of differentiated neurons (H9 control, hom1, and hom2) on D72, co-immunostained for GFP, MAP2, CHD7, and DAPI (yellow arrowheads, axons labeled by GFP but not MAP2; scale bars, 50 μm). **E**, **F** Quantification of total dendrite length (**E**) and branch number (**F**) of differentiated neurons (H9 control, hom1, and hom2) on D72 (25–30 neurons were analyzed per group; *P < 0.05, **P < 0.01, ***P < 0.001, ****P < 0.0001; one-way ANOVA; see also Fig. S3).

During the differentiation of NPCs towards mature neurons, neurites including axons and dendrites start to develop and form functional synapses. Thus, to determine whether neuronal development is affected by an intronic variant of *CHD7*, we measured the neurites growth of differentiated neurons derived from hESCs. To visualize the morphology of neurons, we transfected GFP-expressing plasmids on D38 after neural differentiation. On D43, we performed immunostaining using antibodies against GFP and MAP2 (protein marker for dendrites) and found that the signals of GFP and MAP2 fully overlapped (Fig. 3A), suggesting that the polarity of neurons has not been established as axonal differentiation has not accomplished. Although neuronal differentiation is ongoing on D43, we still found that total neurite length and branch number was lower in neurons carrying homozygous mutations than in wild-type neurons derived from H9 ESCs, although the difference was not significant (Fig. 3B, C).

To determine whether intronic variation of *CHD7* affects mature neurons, we transfected GFP-expressing plasmid into mature neurons derived from human embryonic stem cells on D68 from the initiation of neural differentiation. On D72, we immunostained intronic variant and wild-type neurons, and found that the long axons labeled by GFP could not be marked by MAP2 (Fig. 3D), suggesting that axonal differentiation was finished, and mature dendrites were labeled by MAP2. We found that both dendritic length and branch number were significantly lower in neurons carrying the intronic variant than in wild-type neurons derived from H9 ESCs (Fig. 3E, F), suggesting that the intronic variation of *CHD7* impairs the formation of dendritic morphology.

### The Morphological Defects Caused by Intronic Variation are Rescued by Knocking Down its Up-Regulated Gene *TBR1*

**Fig. 4.**
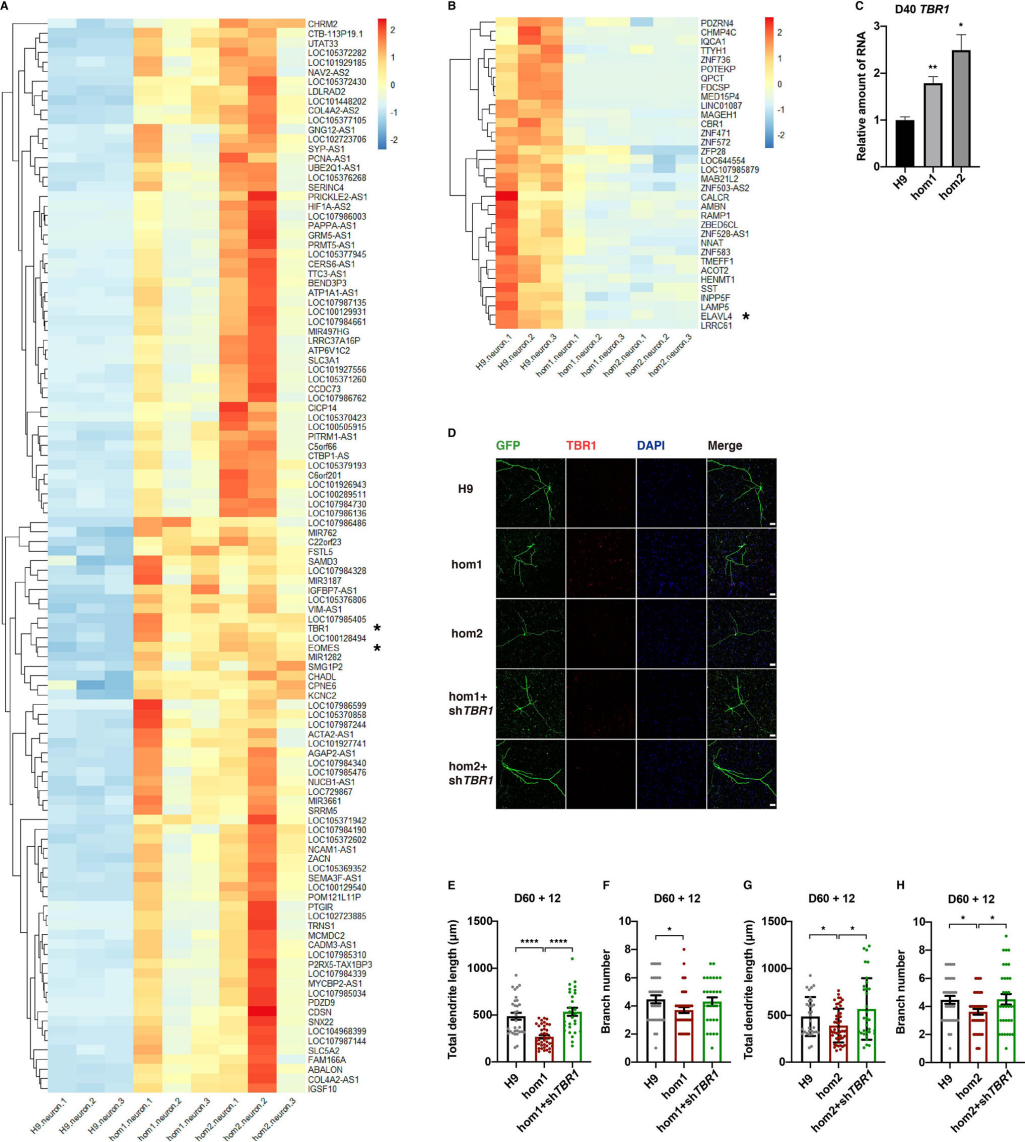
The morphological defects caused by intronic variation are rescued by knocking down its up-regulated gene *TBR1.* **A**, **B** Heatmaps showing up-regulated genes (**A**) and down-regulated genes (**B**) (fold change > 1.5, FDR < 0.05) in differentiated hom1 and hom2 neurons compared to H9 controls on D40. (color scale on right; *genes involved in neural differentiation and development). **C** *TBR1* mRNA levels assessed by RT-qPCR in H9 and cell lines carrying homozygous mutants (hom1 and hom2). Values represent the mean ± SEM (*n* = 3, *P < 0.05, **P < 0.01, ***P < 0.001, ****P < 0.0001; *t*-test). **D** Representative images of differentiated neurons (H9 control, hom1, and hom2) on day D72 transfected with GFP and shRNA, co-immunostained for GFP, MAP2, TBR1, and DAPI (scale bars, 50 μm). **E–H** Quantification of the total dendrite length (**E** and **G**) and branch number (**F** and **H**) of differentiated neurons (H9 control, hom1, and hom2) on D72 (at least 30 neurons were analyzed for each group; *P < 0.05, **P < 0.01, ***P < 0.001, ****P < 0.0001; one-way ANOVA; see also Fig. S4).

To investigate whether the intronic variation of *CHD7* interferes with the gene expression profile during neuronal development, we performed RNA-seq on RNA from differentiated neurons on D40 after the initiation of neural differentiation. Interestingly, we found that the genes that were up-regulated in neurons derived from both hom1-H9-hESCs and hom2-H9-hESCs compared to the wild-type, were much more numerous than down-regulated genes (Figs. 4A, B, S4A), strongly suggesting that CHD7 plays a negative role in regulating gene expression in neurons (genes closely associated with neural development are marked with asterisks in Fig. 4A, B). We further validated these findings using real-time qPCR in neurons on D40 (Figs. 4C, S4B, C). We found that *TBR1*, a critical gene implicated in autism, was strongly up-regulated in neurons carrying the intronic variation, comparing to wild-type neurons (Fig. 4A).

The *TBR1* gene has been implicated in amygdala development and the laminar patterning of retinal ganglion cells as well as cortical neurons [25–27]. Interestingly, TBR1 is a putative transcription factor that is strongly expressed in glutamatergic early-born cortical neurons and regulates differentiation of the preplate and layer VI neurons [28]. Another up-regulated gene *EOMES* (*TBR2*), and *TBR1* are expressed sequentially by intermediate progenitor cells and postmitotic neurons in developing neocortex [29]. Given the function of TBR1 in regulating neuron projection development, as well as the association of several of the regulated genes with TBR1, we considered whether the morphological defects could be improved by restoring the expression of *TBR1*.

We transfected shRNAs for *TBR1* and GFP-expressing plasmid into differentiated neurons with the *CHD7* intronic variant on D60. As controls, shRNAs for *DsRed* and GFP-expressing plasmid were transfected into D60 differentiated wild-type neurons. On D72, we immunostained neurons with anti-GFP (Fig. 4D). From the results, both the dendritic length and branch number in neurons with the intronic variant transfected with shRNA for *TBR1* were improved over those transfected with shRNA for *DsRed*, and did not differ from the wild-type (Fig. 4E–H). This evidence indicated that *TBR1* is an important downstream gene of CHD7.

### Intronic Variation of *CHD7* Affects Alternative Splicing, Resulting in Three Abnormal Transcripts that are Functionally Deficient

The precise excision of introns is catalyzed by a sophisticated ribonucleoprotein machinery called the spliceosome [30]. Intron–exon boundaries are delimited by short consensus sequences at the 5′ (donor) and 3′ (acceptor) splicing sites that are recognized by the spliceosome. In addition, the spliceosome interacts with a catalytic adenosine (the branch point) and a polypyrimidine tract (PyT) located between the branch point adenosine and the 3′ splicing sites [31].

Since the intronic variation of the *CHD7* gene is located adjacent to the branch point and may form a new splicing site, it would be intriguing to determine whether alternative splicing processes are altered in cells carrying the intronic variant.

**Fig. 5.**
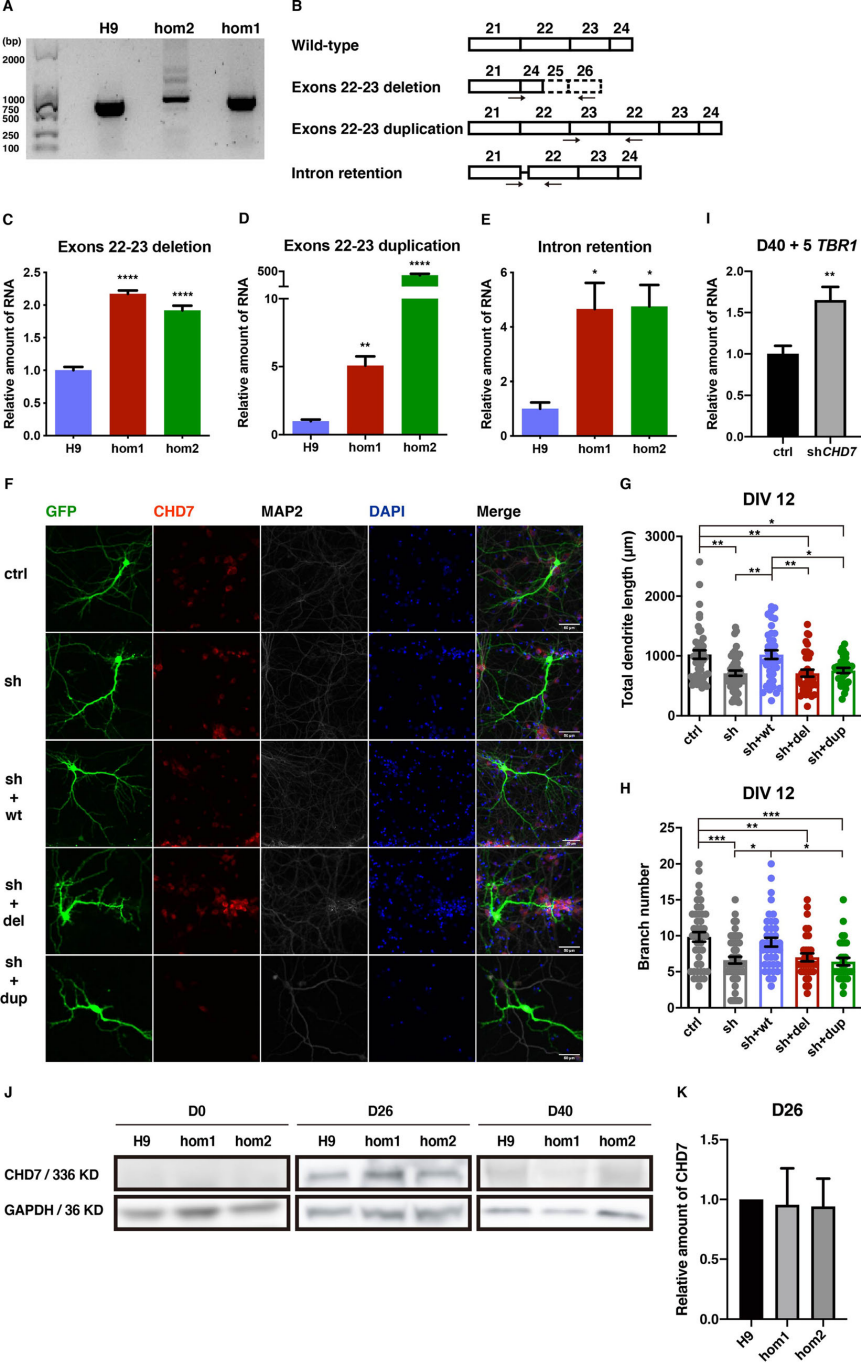
Intronic variation of *CHD7* affects alternative splicing, resulting in three abnormal transcripts that are functionally deficient. **A** Agarose gel electrophoresis illustrating PCR products of exons 21–24 from cDNA of H9 control, hom1, and hom2. **B** Schematic of alternative splicing around the intronic mutation. **C–E** mRNA levels assessed by RT-qPCR for the transcripts with exons 22–23 deletion (**C**), exons 22–23 duplication (**D**), and intron retention (**E**) in H9 control and two point-mutant cell lines. Values represent the mean ± SEM (*n* = 3, *P < 0.05, **P < 0.01, ***P < 0.001, ****P < 0.0001; one-way ANOVA). **F** Representative images of E14.5 mouse primary cortical neurons transfected with GFP, together with shRNA for DsRed as control or shRNA for mouse *Chd7*, and with human *CHD7* transcript wild-type, del or dup at DIV 12, co-immunostained for GFP, CHD7, MAP2, and DAPI (scale bars, 50 μm). **G**, **H** Quantification of total dendrite length (**G**) and branch number (**H**) of DIV 12 neurons (at least 30 neurons were analyzed for each group; *P < 0.05, **P < 0.01, ***P < 0.001, ****P < 0.0001; one-way ANOVA). **I** *TBR1* mRNA levels assessed by RT-qPCR in differentiated neurons after transfection with shRNA for *CHD7* at D40 for 5 days. Values represent the mean ± SEM (*n* = 8, *P < 0.05, **P < 0.01, ***P < 0.001, ****P < 0.0001; *t*-test). **J** Western blot analysis of CHD7 protein expression on D0, D26, and D40 of differentiation. **K** Quantification of CHD7 protein expression on D26 of differentiation. Values represent the mean ± SEM (*n* = 3, *P < 0.05, **P < 0.01, ***P < 0.001, ****P < 0.0001; *t*-test; see also Fig. S5).

Since the intronic variant (ch8-61757392-C-T) was located in the intron between exons 21 and 22 (Fig. 1A), in order to examine potential transcripts, we amplified mRNA segments of *CHD7* from exon 21 to exon 24 with PCR in wild-type hESCs and cells carrying the homozygous intronic variant (Fig. 5A). The amplification products were ligated with the T-vector followed by monoclonal sequencing. Interestingly, we found that, besides wild-type transcripts found in wild-type hESCs, there were three novel transcripts in hESCs carrying the intronic variant: a transcript with deletion of exons 22–23 (del), a transcript with duplication of exons 22–23 (dup), and a transcript with retention of 32 bp of an intronic fragment with seamless connection upstream to exon 22 (Fig. 5B).

To further determine the expression level of each novel transcript in wild-type hESCs and hESCs carrying the intronic variant, we assessed the level of each transcript using real-time qPCR with specific primers in RNA samples from corresponding cell lines (arrows in Fig. 5B). Importantly, we found that the expression levels of the three novel transcripts were significantly higher in hESCs with the intronic variant than in H9 wild-type hESCs (Fig. 5C–E), suggesting that the intronic variant compromises the alternative splicing of *CHD7* mRNA.

In order to further verify that the intronic variant affects the alternative splicing of *CHD7* in other cell lines, we also constructed cell lines with the intronic point mutation using HEK293 cells, and successfully screened heterozygotes and homozygotes including three point mutations (an intronic mutation and two synonymous mutations, abbreviated as 3PM het and 3PM hom, respectively), as well as heterozygotes and homozygotes with only two synonymous mutations (abbreviated as 2PM het and 2PM hom, respectively) as controls. Synonymous mutations were introduced in the exonic sites using the same strategy as in hESCs, to avoid unwanted digestion of Cas9 after homologous recombination. We found that intronic variants led to increases in the three abnormal transcripts in HEK293 cells as well, consistent with findings in hESCs (Fig. S5A–C).

The proportion of each transcript in hESCs and HEK293 cells was analyzed based on the cycle threshold from real-time qPCR (Fig. S5D, E). The dup transcript accounted for a considerable proportion of the total transcripts in hESCs and HEK293 cells. In contrast, the dup transcript was replaced by the del transcript in 3PM het HEK293 cells. The intronic retention transcript took up a very small proportion. These results suggest that the intronic variation regulates the alternative splicing of exons 22–23 by unknown mechanisms, and produces novel splicing forms by changing the selection of splicing sites.

Combined with the results from hESCs and HEK293 cells, the three novel transcripts were significantly increased with intronic variation, but the degree was different in each strain. This may be because the effect of intronic variation is not limited to what we observed, making dominant selection of splicing sites randomly, which in turn further deepens the differences.

To further determine whether these abnormal types of alternative splicing occur in differentiated neural precursors and neurons, we performed real-time qPCR experiments in NPCs and neurons derived from hESCs. Briefly, we amplified mRNA segments of *CHD7* from exon 21 to exon 24 by PCR on D26 and D40 in differentiated wild-type H9 cells and cells carrying homozygous intronic variants. We found that the three types of abnormal transcript were all present in hESCs and consistently, the expression levels of these transcripts were significantly higher in cells with the intronic variant on D26 and D40 than in H9 wild-type cells at the same stage (Fig. S5F–K). Based on these results, we were curious about the existence of the three novel transcripts in the mRNA of variant carriers. Unfortunately, it was difficult to obtain samples from patients.

We then set out to further address whether the three abnormal transcripts of *CHD7* caused by the intronic variant are functional. The transcript containing extra 32 bp of the intronic fragment would have a frameshift during translation, and thus be functionally deficient. Since exons 22 and 23 together contain 360 bp, the del transcript lacking these exons and the dup transcript containing two copies of these exons could be translated to full-length proteins with fewer or more amino-acids encoded by exons 22 and 23. To determine whether the protein products translated from the del and dup transcripts function differently from the wild-type CHD7 protein, we constructed the del and dup CHD7 expression vectors and assessed the expression of wild-type, del and dup CHD7 in HEK293 cells by Western blot. We found that the protein products generated from the del and dup transcripts were comparable to the wild-type CHD7 protein, at ~ 336 kD (Fig. S5L).

To determine whether the proteins carrying duplication of exons 22 and 23 or without exons 22 and 23 have function normally like wild-type CHD7, we knocked down endogenous *Chd7* in cultured cortical neurons from E14.5 mice using shRNA that only targeted mouse *Chd7* and not human *CHD7* (Fig. S5M, N). Meanwhile, we performed rescue experiments, by co-expressing wild-type CHD7, del and dup CHD7 constructs, along with shRNA against *Chd7*.

We first measured axonal growth at 3 days *in vitro* (DIV) and found that the total axonal length decreased significantly after *Chd7* knockdown. This was fully rescued by wild-type human *CHD7*, but not the del or dup form of *CHD7*, suggesting that the del and dup forms of *CHD7* are loss-of-function transcripts (Fig. S5O, P).

At 12 DIV, we found that the dendritic length and branch number significantly decreased after *Chd7* knockdown (Fig. 5F–H), indicating that *Chd7* plays a critical role in the normal development of cortical neurons. Importantly, the defects of dendritic growth were fully rescued by wild-type human *CHD7*, but not the del or dup transcripts (Fig. 5F–H). Together, this evidence demonstrates that the del and dup *CHD7* transcripts caused by the intronic variant act in a loss-of-function manner.

In the previous results, we found that the intronic variant led to an increase of *TBR1* expression (Fig. 4C) and defective neuronal morphology (Fig. 4E–H). In order to investigate whether this was caused by the decrease of CHD7, we analyzed the expression of *TBR1* 5 days after knockdown of *CHD7* by shRNA in differentiated neurons on D40. The results showed that *CHD7* mRNA was knockdown by 40% (Fig. S5Q) and *TBR1* was significant up-regulated (Fig. 5I). It is worth noting that the protein expression of CHD7 was predominantly in neural precursors during differentiation and rarely in hESCs and neurons (Fig. 5J). There was no significant difference in the high expression period on D26 (Fig. 5K), so it may be that the subsequent effect caused by the intronic variant is through *CHD7* mRNA rather than the protein, if the factor that the protein with high molecular weight cannot be quantified accurately using Western blot could be excluded. If this is the case, mRNA accumulation in hESCs and neurons is functional rather than redundant.

Based on the above results, we found that the intronic variant produced three novel transcripts, especially a huge increase of the del transcript in 3PM het of HEK293 cells (Fig. S5A), the dup transcript in 3PM hom of HEK293 cells (Fig. S5B), and in hom2 of H9 cells (Fig. 5D; Fig. S5G, J), indicating that the intronic variant leads to instability of exons 22–23 splicing. Moreover, both transcripts did not function normally, it can be concluded that this further reduced the content of normal transcripts to varying degrees due to alternative splicing. This may partly explain the phenotypic difference of the intronic variant carriers.

Thus, the intronic variant identified in autism patients indeed plays a critical role in regulating the function of *CHD7* by down-regulating the mRNA level and disrupted alternative splicing patterns. The proper neural differentiation and development of human NPCs and neurons were severely affected by the intronic variant, providing crucial evidence supporting the notion that the intronic variant site of *CHD7* is a potential autism susceptibility site.

## Discussion

Genes implicated in autism have provided critical insights into the pathogenesis of the disease. Some of the well-documented autism risk factors include genes associated with rare syndromic forms of ASD (*MECP2*, *FMR1*, and *PTEN*), synaptic cell adhesion and scaffolding molecules (NLGN3, NLGN4, NRXN1, CNTNAP2, and SHANK3), and genes with *de novo* mutations (*CHD8*, *SCN2A*, and *DYRK1A* among others) identified in whole-exome sequencing studies [5]. Due to the high heterogeneity of autism, the phenotype of patients varies from mild to severe and is affected by the genetic background of the family. Therefore, according to the unified criteria for autism diagnosis, large-scale family analysis with family members as controls is very important for the screening of risk mutations. Deleterious mutations are usually screened for in coding regions and splicing sites, because such mutations can result in the loss of gene function. Even so, at most 25% of ASD cases can be shown to have a genetic cause. Understanding of the genetic basis of autism has evolved from high-load disruption caused by a single mutation to a complex of multi-genes\ variation with low-load effects. These mutations alone are not enough to cause the phenotype, but can act as helpers. For this, some carriers become ill while others are asymptomatic, making it difficult to present a comprehensive picture of genetic variation in ASD patients.

Many introns contain highly conserved sequence elements, including the consensus splice site sequences and the binding sites for regulatory proteins, as well as the sequences of non-coding RNA genes [32]. Alternative splicing increases the diversity of the transcriptome by generating multiple mRNA isoforms from a single gene. A pre-mRNA molecule can be alternatively spliced through exon-skipping, alternative splice-site selection, and intron retention [33]. Mutations in intronic regions have been documented in various diseases. For example, a mutation that creates a novel donor splice site leads to the inclusion of a 95-nucleotide intronic sequence in *BRCA2* mRNA [31, 34]. A mutation that creates a novel binding site for SRSF1 activating a splicing enhancer element thus leads to inclusion of a 147-nucleotide pseudo-exon in *COL4A5* mRNA [31, 35]. In addition, genetic variants have been reported to cause disease through inactivation of intron-encoded RNA genes [36]. In our study, intronic variation created a novel acceptor splice site and led to the inclusion of a 32-nucleotide intronic sequence in *CHD7* mRNA (Fig. 5B, E). Interestingly, two abnormal transcripts with deletion of exons 22–23 and duplication of exons 22–23 were also found (Fig. 5B–D). We have not further explored the mechanism of mutation disturbing the splicing stability of exons 22–23. Nevertheless, the results provide a new form of alternative splicing. If exon deletion is caused by the selection of splicing sites and leads to exon-skipping, the mechanism of exon duplication is much more complicated; for example, the sequence repeat process may be a combination of transcription and splicing.

As evidence we present in this study, we found that intronic variation of *CHD7* has critical effects on neural differentiation and morphological development. In our study, transcriptome sequencing was performed in three stages of differentiation (hESCs, NPCs, and neurons). The genes with significant differences between the wild-type H9 cells and those carrying *CHD7* intronic variants on D26 and D40 were listed for analysis (Figs. 2G, H and 4A, B). Few genes were regulated during the ESC period (data not show). Moreover, results showed that *CHD7* mRNA was expressed in hESCs while protein expression was scarce, suggesting that the function of CHD7 in stem cells is limited. In NPCs, the affected genes participate in dopaminergic neuronal differentiation, dentate gyrus development, and the Wnt signaling pathway (Figs. 2G, H, S2B). In neurons, the affected genes are involved in cell metabolism, signal transduction pathways, synaptic transmission, and axon guidance (Figs. 4A, B, S4A).

In a comprehensive analysis of three stages, we found that the up-regulated genes in each period were highly specific and regulated only in the specific period, suggesting that the inhibitory action of *CHD7* on its target genes is stage-specific. Among the down-regulated genes, there were not only highly specific genes, but also genes that were continuously regulated. For example, some genes were down-regulated in stem cells and precursor cells such as *ZNF826P*; some genes were down-regulated in precursor cells and neurons such as *ELAVL4*; and some genes were down-regulated in all three stages such as *LINC01087*, *MED15P4*, and *ZNF528-AS1*. In addition, although some genes were regulated only in a specific period, there were direct interactions between regulated genes in different periods. For example, *FEZF2* that was up-regulated in the precursor phase can be inhibited by *TBR1* that is up-regulated in neurons during neocortical development [37]. Based on the above results, CHD7 begins to accumulate in stem cells, but plays a role mainly in neural differentiation and development. Its function is partly period-specific and partly continuous, which suggests that different treatments should be given at different stages in the development of the defects caused by *CHD7* mutation.

## Conclusions

In conclusion, the level of *CHD7* mRNA significantly decreased in cell lines carrying homozygous mutants compared to control. Upon differentiation towards the forebrain neuronal lineage, neural cells carrying the *CHD7* intronic variant exhibited developmental delay and maturity defects. *TBR1*, a gene also implicated in ASD, significantly increased in neurons carrying the *CHD7* intronic variant. Furthermore, the morphological defects in neurons carrying *CHD7* intronic mutations were rescued by knocking down *TBR1*, indicating that *TBR1* may be responsible for the defects in *CHD7*-related disorders. Finally, the *CHD7* intronic variant generated three abnormal forms of transcripts through alternative splicing, all of which exhibited loss-of-function in functional assays. Our study provides crucial evidence to support the notion that the intronic variant site of *CHD7* is a potential autism susceptibility site, shedding new light on identifying the functions of intronic variants in genetic studies of autism.

## Supplementary Information

Below is the link to the electronic supplementary material.

Supplementary file1 (PDF 1929 KB)
