# Supplementary material for: An Intronic Variant of CHD7 Identified in Autism Patients Interferes with Neuronal Differentiation and Development
Source: Neurosci Bull. 2021 May 4;37(8):1091–106. doi: 10.1007/s12264-021-00685-w (PMC8353028; doi:10.1007/s12264-021-00685-w)
Supplement: Supplementary file 1 — Supplementary file1 (PDF 1929 KB) [file 12264_2021_685_MOESM1_ESM.pdf]

Supplementary Materials

Supplementary Figures

A

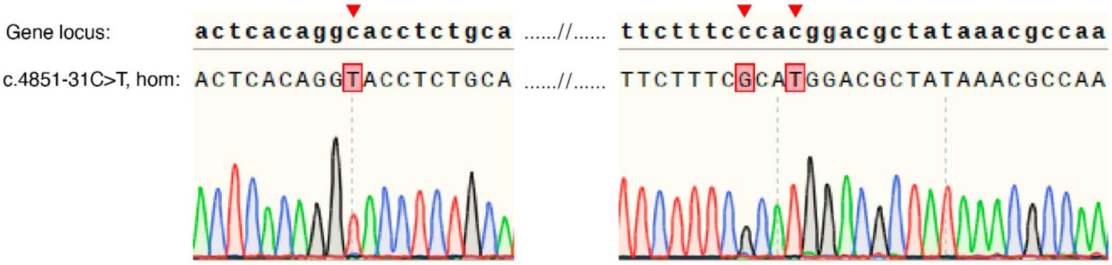

B

Sequence	PAM	Score	Chromosome	Strand	Position	Mismatches
TTGGAGTTAAAAGCGTCCTT	GAG	0.193167	chr5	1	7081648	4
TTGTCTTTTATAGCGTCCTA	AGG	0.173688	chr2	-1	117679569	4
TTGGGGTTTAAGGCCTCCGT	AGG	0.116908	chr6	-1	100455661	4
TTGGCGTTTCCAGCGTCCCC	CAG	0.114448	chr1	1	1986592	4
TTGGCCTTTACTGCGTCCTT	GGG	0.090433	chr1	1	93722298	4

Fig. S1 Identification of cell lines with point mutation. **A** Sequencing of cell lines carrying homozygous mutants of hESCs (red arrowheads, introduced mutant bases). **B** Predicted high-risk off-target sites to be validated. PAM, protospacer adjacent motif.

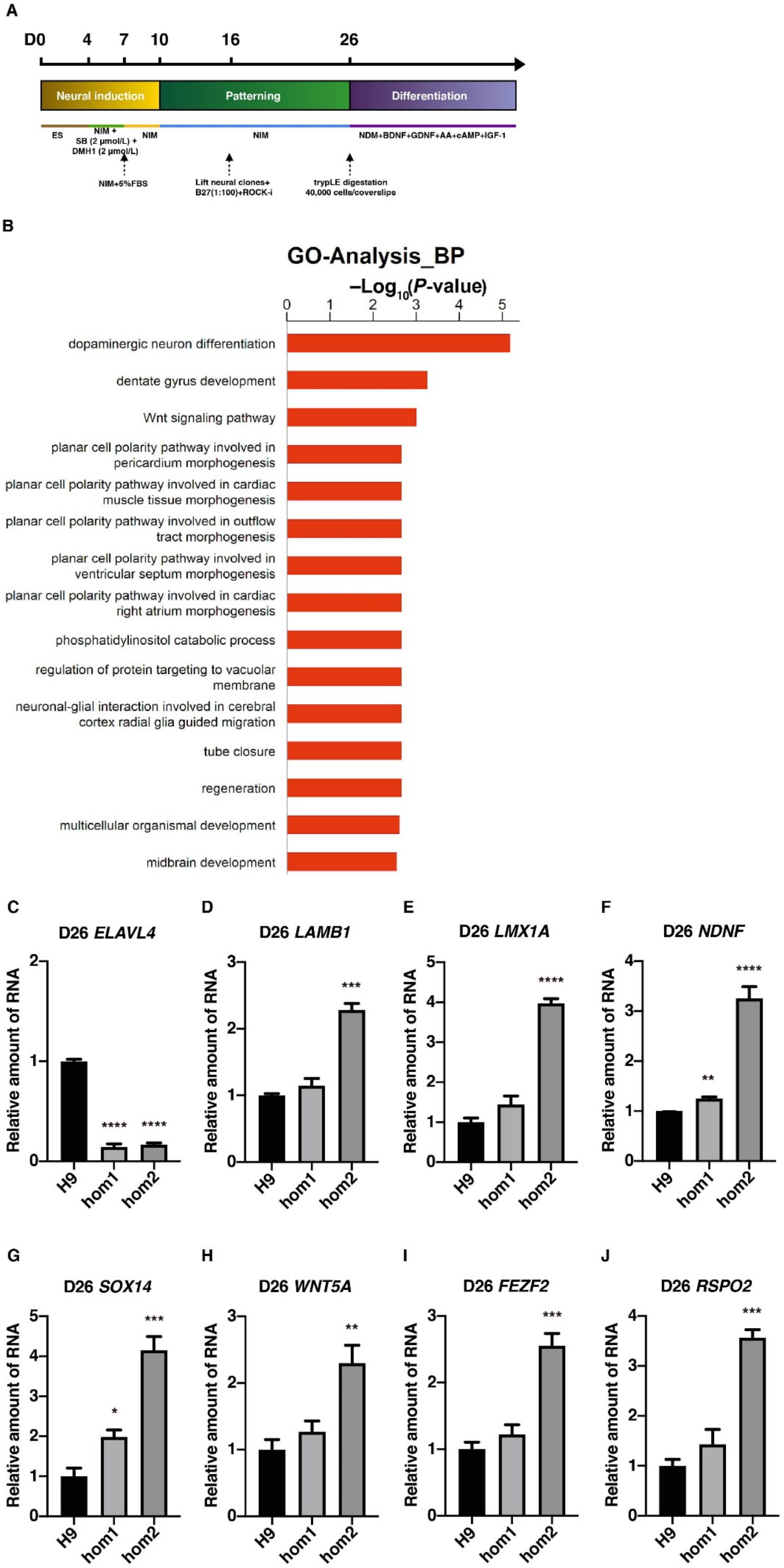
A
D0
4
7
10
16
26
Neural induction
Patterning
Differentiation
ES
NIM +
SB (2 μmol/L) +
DMH1 (2 μmol/L)
NIM
NIM
NDM+BDNF+GDNF+AA+cAMP+IGF-1
NIM+5%FBS
Lift neural clones+
B27(1:100)+ROCK-i
trypLE digestation
40,000 cells/coverslips
B
GO-Analysis_BP
–Log10(P-value)
0 1 2 3 4 5
dopaminergic neuron differentiation
dentate gyrus development
Wnt signaling pathway
planar cell polarity pathway involved in pericardium morphogenesis
planar cell polarity pathway involved in cardiac muscle tissue morphogenesis
planar cell polarity pathway involved in outflow tract morphogenesis
planar cell polarity pathway involved in ventricular septum morphogenesis
planar cell polarity pathway involved in cardiac right atrium morphogenesis
phosphatidylinositol catabolic process
regulation of protein targeting to vacuolar membrane
neuronal-glial interaction involved in cerebral cortex radial glia guided migration
tube closure
regeneration
multicellular organismal development
midbrain development
C
D26 ELAVL4
D
D26 LAMB1
E
D26 LMX1A
F
D26 NDNF
G
D26 SOX14
H
D26 WNT5A
I
D26 FEZF2
J
D26 RSPO2
Relative amount of RNA
H9
hom1
hom2
****
***
**
*

Fig. S2 Effect of intronic variation of *CHD7* on biological processes during differentiation. **A** Timeline of differentiation of hESCs to dorsal forebrain glutamate neurons (ES, human embryonic stem cell; NIM, neural induction medium; SB, TGF-β/Smad inhibitor SB-431542; DMH1, BMP inhibitor dorsomorphin homologue 1; ROCK-i, ROCK pathway inhibitor; NDM, neural differentiation medium; AA, ascorbic acid). **B** Gene Ontology Biological Process analysis of genes regulated in differentiated neural precursor hom1 and hom2 cells compared to H9 controls on D26 [GO terms with Benjamini-adjusted *P*-values <0.05 (Fisher's exact test)]. **C–J** mRNA levels assessed by RT-qPCR for genes closely related to neural differentiation and development identified in RNAseq in cell lines carrying homozygous mutants on D26. Value represents the mean ± SEM (n = 3, *P < 0.05, **P < 0.01, ***P < 0.001, ****P < 0.0001; t-test).

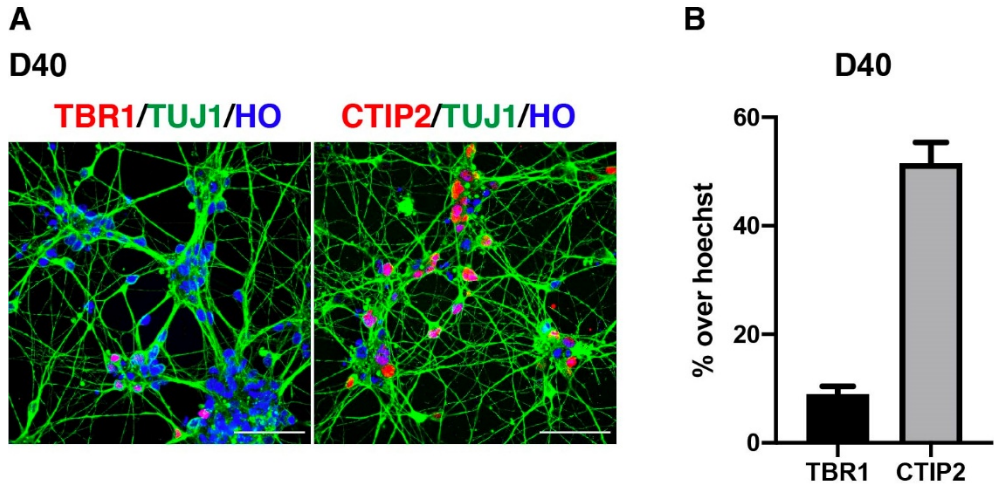

Fig. S3 Identification of differentiated neurons. **A** Representative images of differentiated H9 neurons on D40 co-immunostained for regionalization markers of the dorsal forebrain (TBR1 and CTIP2) with TUJ1 and Hoechst (HO) (scale bars, 50 µm). **B** Percentage of total cells expressing neuronal regionalization markers from D40 of differentiation (~300 cells in 8 fields were analyzed for each group).

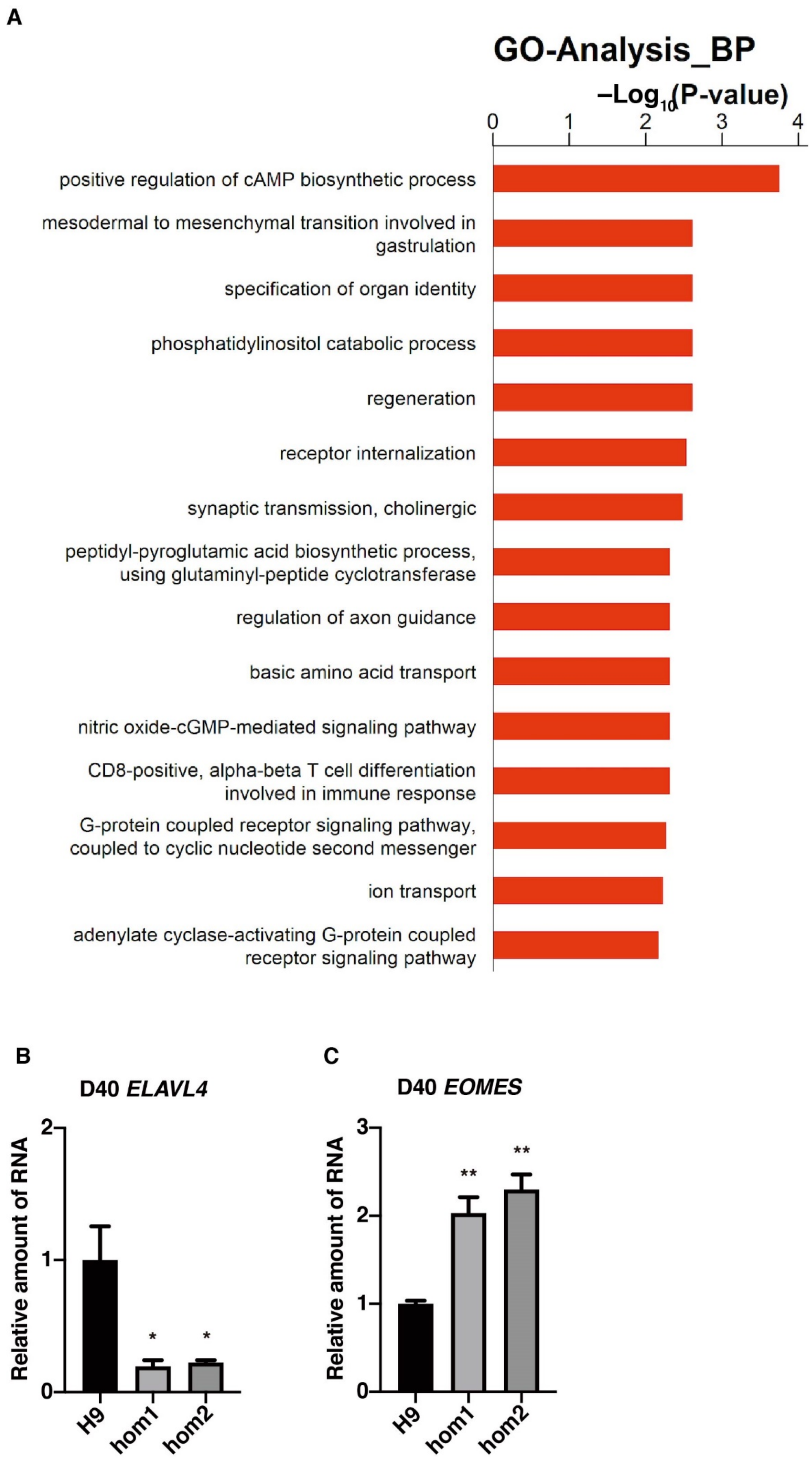

A
GO-Analysis_BP
–Log10(P-value)
0
1
2
3
4
positive regulation of cAMP biosynthetic process
mesodermal to mesenchymal transition involved in gastrulation
specification of organ identity
phosphatidylinositol catabolic process
regeneration
receptor internalization
synaptic transmission, cholinergic
peptidyl-pyroglutamic acid biosynthetic process, using glutaminyl-peptide cyclotransferase
regulation of axon guidance
basic amino acid transport
nitric oxide-cGMP-mediated signaling pathway
CD8-positive, alpha-beta T cell differentiation involved in immune response
G-protein coupled receptor signaling pathway, coupled to cyclic nucleotide second messenger
ion transport
adenylate cyclase-activating G-protein coupled receptor signaling pathway
B
D40 ELAVL4
Relative amount of RNA
2
1
0
*
*
H9
hom1
hom2
C
D40 EOMES
Relative amount of RNA
3
2
1
0
**
**
H9
hom1
hom2

Fig. S4 Effect of intronic variation of *CHD7* on biological processes during neurodevelopment. **A** Gene Ontology Biological Process analysis of genes regulated in differentiated hom1 and hom2 neurons compared to H9 controls on D40 [GO terms with Benjamini adjusted *P*-values < 0.05 (Fisher's exact test)]. **B, C** mRNA levels assessed by RT-PCR for genes closely related to neural differentiation and development identified in RNAseq in cell lines carrying homozygous mutants (hom1 and hom2) on D40. Values represent the mean ± SEM ($n = 3$, **P* < 0.05, ***P* < 0.01, ****P* < 0.001, *****P* < 0.0001; *t*-test).

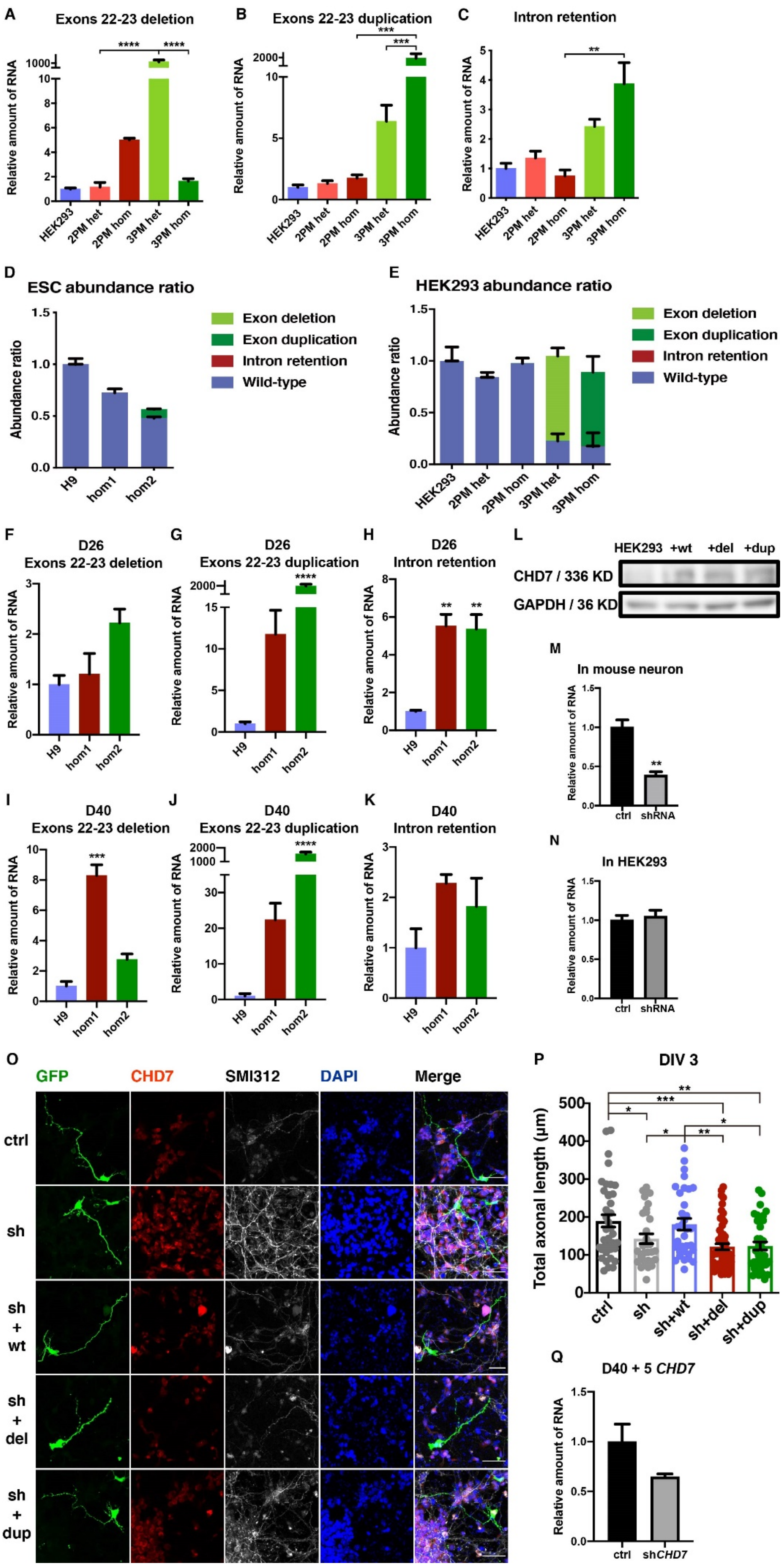

A
Exons 22-23 deletion
Relative amount of RNA
HEK293
2PM het
2PM hom
3PM het
3PM hom
B
Exons 22-23 duplication
C
Intron retention
D
ESC abundance ratio
Abundance ratio
H9
hom1
hom2
Exon deletion
Exon duplication
Intron retention
Wild-type
E
HEK293 abundance ratio
F
D26
Exons 22-23 deletion
G
D26
Exons 22-23 duplication
H
D26
Intron retention
I
D40
Exons 22-23 deletion
J
D40
Exons 22-23 duplication
K
D40
Intron retention
L
HEK293 +wt +del +dup
CHD7 / 336 KD
GAPDH / 36 KD
M
In mouse neuron
ctrl
shRNA
N
In HEK293
O
GFP
CHD7
SMI312
DAPI
Merge
ctrl
sh
sh + wt
sh + del
sh + dup
P
DIV 3
Total axonal length (μm)
ctrl
sh
sh+wt
sh+del
sh+dup
Q
D40 + 5 CHD7
ctrl
shCHD7

Fig. S5 Expression and function of abnormal transcripts. **A–C** mRNA levels assessed by RT-qPCR for transcripts with exons 22–23 deletion (**A**), exons 22–23 duplication (**B**), and intron retention (**C**) in point mutant HEK293 cells, grouped into HEK293 cell controls, synonymous mutation controls (2PM het and 2PM hom), and intronic mutations (3PM het and 3PM hom). Values represent the mean ± SEM (n = 3, *P < 0.05, **P < 0.01, ***P < 0.001, ****P < 0.0001; one-way ANOVA). **D, E** Relative abundance of each transcript in hESCs (**D**) and HEK293 cells (**E**). **F–K** mRNA levels assessed by RT-qPCR for transcripts in differentiated NPCs on D26 (**D–F**) and neurons on D40 (**G–I**). Values represent the mean ± SEM (n = 3, *P < 0.05, **P < 0.01, ***P < 0.001, ****P < 0.0001; one-way ANOVA). **L** Western blots of CHD7 protein expression using HEK293 cells transfected with wild-type transcript, exons 22–23 deletion transcript, and exons 22–23 duplication transcript. **M**, **N** mRNA levels assessed by RT-qPCR for CHD7 in E14.5 mouse primary cortical neurons (**K**) and HEK293 cells after transfection with shRNA for mouse *Chd7* for 4 days (**L**). Values represent the mean ± SEM (n = 3, *P < 0.05, **P < 0.01, ***P < 0.001, ****P < 0.0001; t-test). **O** Representative images of E14.5 mouse primary cortical neurons transfected with GFP, together with shRNA for *DsRed* as control or shRNA for mouse *Chd7*, and with human *CHD7* transcript wt, del or dup at DIV 3, co-immunostained for GFP, CHD7, SMI312, and DAPI (scale bars, 50 μm). **P** Quantification of total axonal length in DIV 3 neurons (at least 30 neurons were analyzed for each group; *P < 0.05, **P < 0.01, ***P < 0.001, ****P < 0.0001; one-way ANOVA). **Q** mRNA levels assessed by RT-qPCR for *CHD7* in differentiated neurons after transfection with shRNA for *CHD7* at D40 for 5 days. Values represent the mean ± SEM (n = 8, *P < 0.05, **P < 0.01, ***P < 0.001, ****P < 0.0001; t-test).
